# Amino Acids Metabolism in Retinopathy: From Clinical and Basic Research Perspective

**DOI:** 10.3390/metabo12121244

**Published:** 2022-12-09

**Authors:** Mengxue Xia, Fang Zhang

**Affiliations:** 1 National Clinical Research Center for Eye Diseases, Shanghai General Hospital, Shanghai Jiao Tong University School of Medicine, Shanghai 200080, China; 2 Shanghai Key Laboratory of Ocular Fundus Diseases, Shanghai 200080, China; 3 Shanghai Engineering Center for Visual Science and Photomedicine, Shanghai 200080, China; 4 Shanghai Engineering Center for Precise Diagnosis and Treatment of Eye Diseases, Shanghai 200080, China

**Keywords:** retinopathy, metabolomics, amino acids, diabetic retinopathy, age-related macular degeneration, retinopathy of prematurity, vision impairment

## Abstract

Retinopathy, including age-related macular degeneration (AMD), diabetic retinopathy (DR), and retinopathy of prematurity (ROP), are the leading cause of blindness among seniors, working-age populations, and children. However, the pathophysiology of retinopathy remains unclear. Accumulating studies demonstrate that amino acid metabolism is associated with retinopathy. This study discusses the characterization of amino acids in DR, AMD, and ROP by metabolomics from clinical and basic research perspectives. The features of amino acids in retinopathy were summarized using a comparative approach based on existing high-throughput metabolomics studies from PubMed. Besides taking up a large proportion, amino acids appear in both human and animal, intraocular and peripheral samples. Among them, some metabolites differ significantly in all three types of retinopathy, including glutamine, glutamate, alanine, and others. Studies on the mechanisms behind retinal cell death caused by glutamate accumulation are on the verge of making some progress. To develop potential therapeutics, it is imperative to understand amino acid-induced retinal functional alterations and the underlying mechanisms. This review delineates the significance of amino acid metabolism in retinopathy and provides possible direction to discover therapeutic targets for retinopathy.

## 1. Introduction

Vision impairment declines the life quality of patients to a large extent, affecting their economic and educational opportunities and even threatening their lives [1,2,3]. According to the estimation of the World Health Organization in 2020, 43.3 million people were blind and 295 million people had moderate and severe vision impairment [4]. Among all the diseases which cause vision impairment, retinopathy comprises a notable proportion [5]. Retinopathy is defined as the disease of the retina and it includes diabetic retinopathy (DR), age-related macular degeneration (AMD), retinopathy of prematurity (ROP), hypertensive retinopathy (HR), and others such as autoimmune retinopathy (AIR). A large number of people are suffering from various retinopathy, ranging from infants, children, the working-age populations to seniors. Early diagnosis and timely therapy of retinopathy is an utmost need.

Clinically, the main diseases of retinopathy have their processes and features. Diabetic retinopathy (DR) is one of the most common complications of diabetes and remains the leading cause of preventable blindness in working-aged people. It is identified in a third of people with diabetes [6]. DR can be classified as non-proliferative diabetic retinopathy (NPDR) and proliferative diabetic retinopathy (PDR) based on the presence of visible ophthalmological changes and the manifestation of retinal neovascularization [7,8]. Meanwhile, age-related macular degeneration (AMD) is the leading cause of incurable blindness worldwide in the elderly. It is classified as an early stage to advanced AMD. Most visual loss occurs in the advanced stages of the disease due to one of two processes: neovascular (“wet”) age-related macular degeneration and geographic atrophy (“late dry”) [9,10]. Similarly, retinopathy of prematurity (ROP) is a proliferative retinal vascular disease affecting the retina of premature infants, whose clinical spectrum varies from spontaneous regression to bilateral retinal detachment and the following total blindness [11]. It affects the life quality of patients from a very early age. Taken together, retinopathy requires diagnostic biomarkers and therapeutic approaches.

Retinopathy is typically detected by fundus examination in clinical practice. Although we have made some progress in the treatment of retinopathy, the limitation of clinical and biochemical detection delays the diagnosis as well as the subsequent treatment [12]. Retinopathy is often diagnosed in the late stage when vision is damaged and therapy options are limited. For the treatment of retinopathy, ocular therapy includes laser photocoagulation, vitrectomy, as well as anti-VEGF therapy. Meanwhile, laser therapy is inherently destructive, associated with unavoidable side effects, and not universally effective in the reversal of visual loss. Additionally, the use of intraocular administration of anti-VEGF agents and corticosteroids in selected eyes also has resistance problems and side effects [6,9]. Therefore, alternative biomarkers and therapies are highly expected.

Retinopathy is highly associated with metabolic alterations. Metabolomics, as a powerful tool to discover potential diagnostic biomarkers and therapeutic targets for diseases [13], enables the detailed characterization of metabolic phenotypes and derangements that underlie diseases since metabolites are the ultimate products of gene, mRNA, and protein activity [14]. Metabolites could convey signals from genetic structure and environment as well as provide functional readouts of physiological states. Metabolomics analysis has been carried out among DR, AMD, and ROP globally and systematically reflected the abnormal metabolic changes by comparing the small molecule composition of various intraocular and blood samples from the patients, mammals, organs, and tissues [15].

Among the studies, amino acids as a type of metabolite drew our attention, because many studies have demonstrated the association between the metabolism of amino acids and retinopathy. Meanwhile, amino acids are of great importance to human beings as the foundation of protein in the body. They are essential for the synthesis of tissue, specific proteins, hormones, enzymes, and neurotransmitters. Amino acids are also involved in energy metabolism via gluconeogenesis and in the regulation of numerous metabolic pathways [16]. In this review, we aim to summarize the latest advances in amino acids in DR, AMD, and ROP based on metabolomics studies.

## 2. Metabolomics-Based Amino Acid Metabolism in Retinopathy

As metabolomics-based technology has the potential for the early diagnosis and even therapy management of retinopathy, several studies explore retinopathy biomarkers and related metabolic pathways in both humans and animal retinopathy models with intraocular and systemic changes.

### 2.1. Amino Acids in Retinopathy by Analysis of Metabolomics

**Figure 1:**
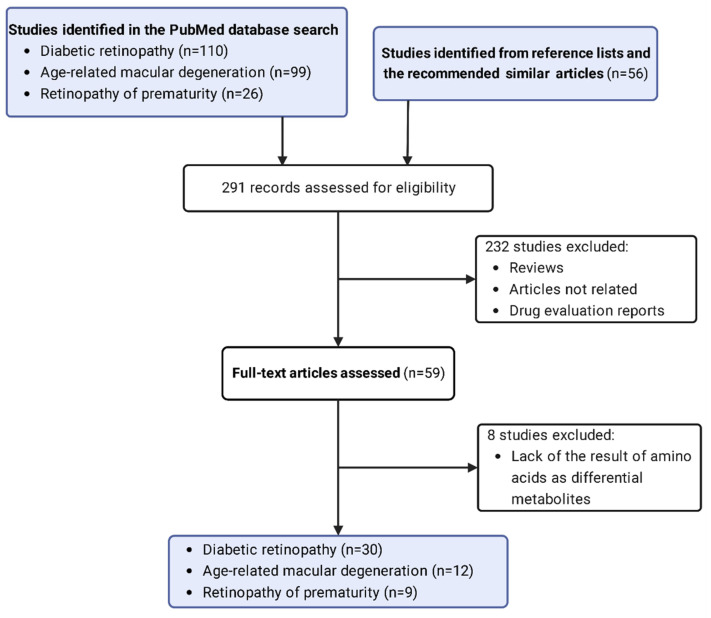
Flow diagram of literature search and study selection for amino acid-based metabolomics of three types of the retinopathy. Studies were searched in the PubMed database with keywords, and 291 records are assessed for eligibility. A total of 232 records remained after exclusion of reviews and articles that are not highly related. Finally, 51 full-text articles with differential amino acids were analyzed in this review including thirty related to diabetic retinopathy, twelve to age-related macular degeneration, and nine to retinopathy of prematurity, respectively.

In recent years, an increasing number of studies have applied metabolomics in DR, AMD, and ROP with certain results. We retrieved relevant articles by searching the PubMed database before September 2022 with the following search terms: (“metabolomics” or “metabonomics” or “metabolome” or “metabolic profiling”) AND (“diabetic retinopathy” or “age-related macular degeneration” or “retinopathy of prematurity”). Additional articles were identified by searching the reference lists of the already-included studies and sometimes the item “similar articles”. After collecting the literature, we selected the results concerning amino acids, which means the specific metabolite belongs to the category of amino acid or is produced by easy reactions with amino acids being the substrates. We included metabolomics studies mainly based on mass spectrometry (MS) or nuclear magnetic resonance (NMR), focusing on studies relating to the three retinal diseases and searching for altered metabolites, excluding review articles and abstracts without full text, resulting in an inclusion of fifty-one studies, including thirty studies of DR, twelve studies of AMD, and nine studies of ROP. A simplified flow diagram of the literature selection is presented (Figure 1). Important details about the study design and methods were extracted from selected articles and summarized in tables (Table 1, Table 2 and Table 3).

Generally, the studies were published in a period of fourteen years since 2009. Factors such as age, gender, or the duration of disease affect metabolic processes, with the ROP study recruiting newborns as study participants and the other two recruiting a middle-aged group and the elderly as participants. Here shows the details.

By focusing on each disease, we could get more detailed information such as the design and techniques in each study. Firstly, in the analysis of DR, thirty studies contain metabolites that are amino acids themselves or simply produced after direct reactions of amino acids such as the addition of methyl group (Table 1). As for the studied species, twenty-eight of them are from human samples, ranging from peripheral blood (plasma and serum) to intraocular fluids (vitreous humor and aqueous humor), and to other samples (retinal tissues, fecal samples, urine, and cerebrospinal fluids). Samples in the remaining two studies are urine from rats and the whole body of 50 pdx1^−/−^ zebrafish, which is a model for diabetic retinopathy lacking the transcription factor pdx1. Each type of sample has its characteristics and advantages. Due to its easier availability and lower invasiveness, peripheral blood is the most commonly used sample and provides a global metabolomic picture [63]. Both serum and plasma can be obtained from blood, with the main difference being the presence or absence of clotting factors [64]. In terms of impact on metabolite detection, plasma appears to have better reproducibility, with serum having higher concentrations [65]. Intraocular fluids including vitreous humor and aqueous humor can directly reflect the metabolic variations in eyes. However, they also have disadvantages. For instance, the vitreous humor, which is a highly aqueous eye fluid and interfaces with the retina, can only be obtained from subjects with PDR during surgery such as a vitrectomy, leading to the absence of vitreous samples of NPDR. In addition, fecal samples can reflect alterations of fecal metabolome and gut microbiota composition, linking DR to the gut metabolome and microbiota–gut–retina axis [66], which has been studied more in recent years along with the development of research on gut microbiota. Various samples are used to study the metabolism of DR, each of which has its characteristics.

As for the metabolomics analysis platform, two main tools are adopted, which are nuclear magnetic resonance (NMR) spectroscopy and mass spectrometry (MS). In our collection, nine studies are carried out using the former while forty use the latter. NMR spectroscopy can be applied to biological samples in various states including liquid, solid, and gaseous samples [67]. The most widely applied NMR technique is the proton NMR method [68]. One significant advantage of NMR is the small number of samples required [15]. MS is often used in tandem with liquid chromatography (LC) or gas chromatography (GC), which are techniques applied to separate metabolites. In particular, LC-MS has been widely used in recent years with much better sensitivity than NMR, allowing it to measure a wider range of metabolites [15]. Overall, the use of NMR and MS has greatly facilitated the development of metabolomics as well as helped scientists to figure out the underlying pathological mechanisms of diseases. The method of sample-collecting and adoption of the platform in DR studies are feasible and reasonable, and there are similar results in the studies of AMD and ROP.

As for AMD, twelve studies focus on amino acid-relating contents. In terms of studied species, only human samples are included (Table 2). Nine of them analyzed the plasma of patients as samples while serum, aqueous humor, and urine samples share the remaining three. NMR and MS are the platforms of metabolomics analysis.

Finally, in the studies of ROP, there are nine studies (Table 3), which use NMR and MS as metabolomics analysis platforms. Besides plasma from humans being the samples in four studies and serum in one study, the heel blood of patients and samples from oxygen-induced retinopathy (OIR) mice and rats are also important objects. Considering the particularity of ROP patients, we only harvested the peripheral blood and avoid injury in the eye. Under this circumstance, animal models show great importance. As a commonly recognized animal model used to study retinal neovascular diseases in vivo, the OIR model is established by exposing newborn pups to a hyperoxia environment [69,70], which has been considered similar to the pathological process of ROP [71]. The studies in ROP include both humans and animals, which is a big advantage.

**Figure 2:**
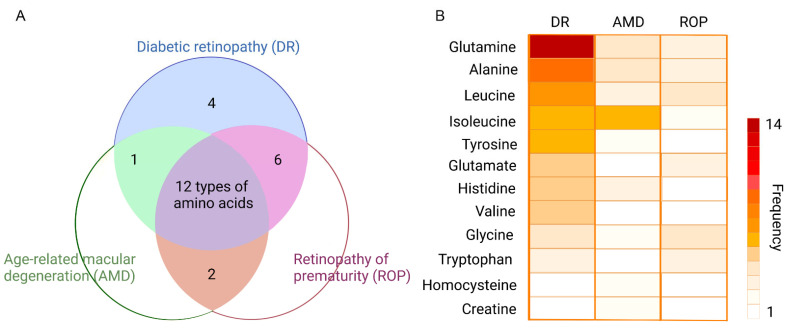
Top-ranking amino acids in retinopathy. (**A**) displays the Venn diagram of the differential amino acids identified in DR, AMD, and ROP, respectively. Twelve amino acids appeared in all three types of the retinopathy. (**B**) The twelve amino acids in (**A**) are collected and presented using a heatmap indicating the reported times in each disease. Accordingly, the more frequently metabolites are reported in metabolomics studies, the darker the color appears in the metabolite zone.

To gain an insight into specific amino acids in retinopathy, we make a Venn diagram to display a count of different amino acids under three main types of the retinopathy (Figure 2A). The three circles represent the three diseases, namely diabetic retinopathy (DR), age-related macular degeneration (AMD), and retinopathy of prematurity (ROP). The color-filled parts, with corresponding numbers, mean how many types of amino acids have been altered in the analysis of metabolomics. For example, in the most central part colored purple, there are twelve types of amino acids altered in the metabolomics analysis of DR, AMD, and also ROP. This Venn diagram shows us directly what needs the most attention. To obtain more information, we flatten the twelve amino acids repeated in three retinal diseases, with their names being displayed at the left of the diagram (Figure 2B). The heatmap is established to indicate the number of reported studies related to the metabolites in AMD/DR/ROP. Accordingly, the more frequently metabolites are reported in metabolomics studies, the darker the color appears in the metabolite zone. Taking glutamine as an example, there are 30, 12, and 9 metabolomics studies for DR, AMD, and ROP, respectively, of which 14 for DR, 4 for AMD, and 3 for ROP are related to glutamine, respectively. Thus, we could see the darker color of glutamine under DR condition compared to AMD and ROP. Though the frequency varies a lot, the proportion does not differ largely. It is clear that the alterations of glutamine and alanine take place frequently in the disorder of DR, AMD, and ROP.

### 2.2. Amino Acids- Related Metabolic Pathways in Retinopathy

**Figure 3:**
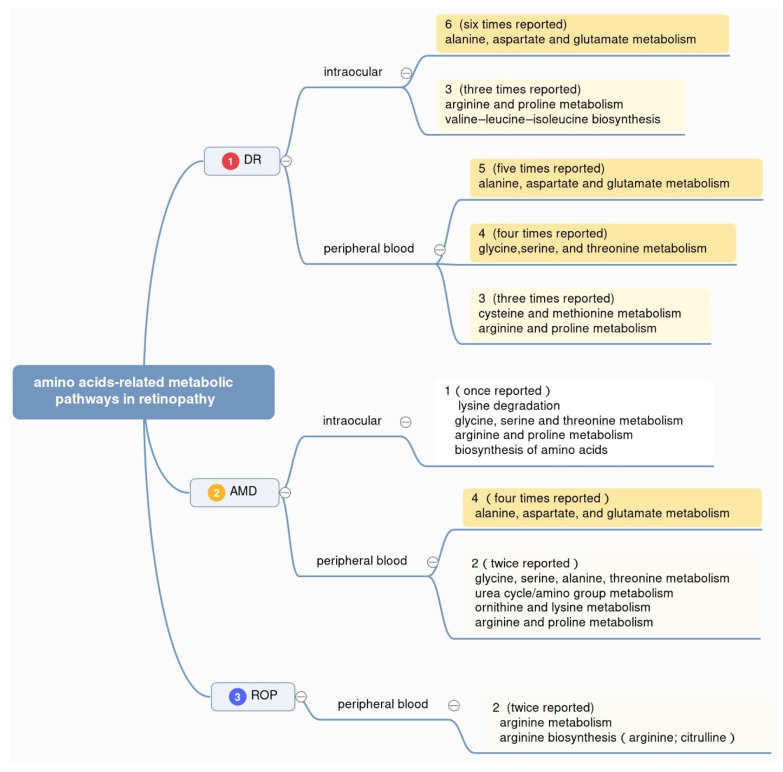
Amino acid-related metabolic pathways in three types of retinopathy. The figure displays the top 30% enriched pathways associated with amino acids in retinopathy, ranked by the reported frequency with different colors.

To gain an in-depth understanding of the mechanism underlying metabolic disorders in retinopathy, the metabolic pathways reported in existing metabolomics studies are also counted, with samples ranging from peripheral blood (plasma and serum) to intraocular fluids (vitreous humor and aqueous humor) of DR, AMD, and ROP patients. Details about altered metabolic pathways are summarized (Figure 3). As shown, we counted the reported times of different pathways in three retinal diseases, respectively. Considering the fact that many metabolic pathways appear only once but make the statistics sophisticated, we make a screening standard, following which the figure only contains part of the statistics. Those in the top 30% of the pathways ranked by reported frequency in each disease are shown.

Top-ranking differentially altered metabolic pathways in DR, AMD, and ROP were summarized in Figure 3. Overall, the pathway of alanine, aspartate, and glutamate metabolism is reported the most, with six times in intraocular fluids from DR patients, five times in peripheral blood from DR patients, and four times in peripheral blood from AMD patients, respectively. At the same time, arginine and proline metabolism and valine–leucine–isoleucine biosynthesis appear in the intraocular humor of DR patients. Glycine, serine, and threonine metabolism shows notable appearance in plasma or serum from DR patients. This result helps us know better about the metabolism of amino acids in retinopathy in the aspect of metabolic pathways.

The pathway of alanine, aspartate, and glutamate metabolism ranked top for its high frequency in the studies of DR and AMD. In the studies of DR patients, alanine, aspartate, and glutamate metabolism is enriched in intraocular samples including the aqueous humor of DR patients using nuclear magnetic resonance (NMR) [37], the vitreous humor of proliferative DR patients by ultra-high-performance liquid chromatography–mass spectrometry (UHPLC-MS) [34], and the vitreous humor of proliferative DR patients via UPLC-MS studies [23]. Interestingly, this pathway also appears frequently in the studies of peripheral blood of DR patients. To be specific, Zhu et al. used liquid chromatography–mass spectrometry (LC-MS) to identify the differential metabolites as well as the enrichment of alanine, aspartate, and glutamate metabolism pathway in the plasma of proliferative DR patients compared to diabetes patients without DR [19]. Yousri et al. observed the differential levels of glutamine as well as the enrichment of alanine, aspartate, and glutamate metabolism pathway in the serum of DR patients compared to diabetes patients without DR [29]. Rhee et al. used ultra-performance liquid chromatography–mass spectrometry (UPLC-MS) and gas chromatography–mass spectrometry (GC-MS) to reveal the similar changes in the plasma of proliferative DR patients compared to control [18].

In several studies of AMD, alanine, aspartate, and glutamate metabolism pathway also clustered in several peripheral blood-based metabolomics studies. Kersten et al. utilized microLC-MS to identify the differential levels of glutamine as well as the enrichment of alanine, aspartate, and glutamate metabolism pathway in the serum of AMD patients and healthy people [52]. Laíns et al. and Osborn et al. utilized ultra-high-performance liquid chromatography–mass spectrometry (UHPLC-MS) and liquid chromatography with Fourier-transform mass spectrometry (LC-FTMS), respectively, and found the differential levels of glutamine, and clustered alanine, aspartate, and glutamate metabolism pathway in the plasma of AMD patients and healthy people [46].

In the studies of ROP, both human samples and animal samples involve the alanine, aspartate, and glutamate metabolism pathway, which is not shown in Figure 3. In detail, Lu et al. chose gas chromatography mass spectrometry (GC-MS) for the plasma of four rat groups with different treatments to mimic the ROP condition [62], Zhou et al. used high-performance liquid chromatography–mass spectrometry (HPLC-MS) in the retina of both eyes of oxygen-induced retinopathy (OIR) mice to identify the differential metabolites and the enrichment of alanine, aspartate, and glutamate metabolism pathway [61]. These studies suggest that alanine, aspartate, and glutamate metabolism pathway may be of high association with retinopathy, especially DR, AMD, and ROP.

### 2.3. Potential Effects of Metabolites in Retina

Among differential metabolites and metabolic pathways related to amino acids, several of them ranked top for their high frequency of appearing in all three types of retinopathy, including glutamine, alanine, and leucine. Here shows the details of some amino acids and retinal cells. Becker et al. [72] analyzed mRNA and miRNA expression profiles of 80 human post-mortem retinal samples from 43 patients diagnosed with various stages of DR. Integrating human retinal single cell RNA sequencing data revealed a continuous loss of retinal ganglion cells (RGC), and Müller cell mediated changes in histidine and β-alanine signaling. In terms of glutamine, it can transform into glutamate relatively easily, and both of them show notable changes as metabolites in the three retinal diseases. We tend to pay more attention to these two amino acids. Glutamate is essential not only as a nutrition supplement but also as a neurotransmitter. Glutamate is also the predominant excitatory amino acid in many regions of the central nervous system (CNS), including the retina. What is more, the retinal ganglion cells (RGCs), as a population of CNS neurons with their soma in the inner retina and axons in the optic nerve, connect the brain and the eyes and allow many studies related to the CNS to be applied in the area of ophthalmology. When it comes to its effects as metabolites in the retina, more studies are being conducted.

**Figure 4:**
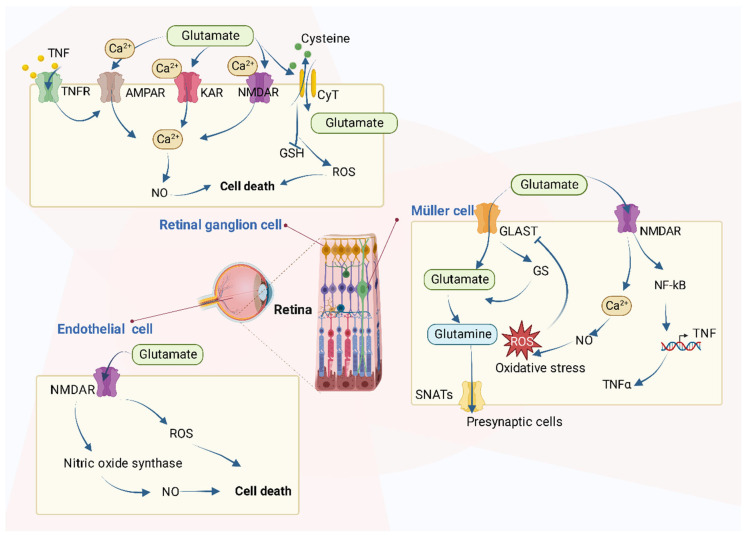
The roles of glutamate on retinal cells. Several types of cells are referred to, including retinal ganglion cells (RGCs), Müller cells, and retinal endothelial cells. In RGCs, excessive glutamate triggers intracellular metabolism changes such as the increase of intracellular Ca^2+^ levels through the over-activation of various receptors and causes cell death. In retinal endothelial cells, glutamate increases both NO and oxidative stress. In Müller cells, glutamate causes oxidative stress and increases the production of TNF-α.

It is reported that the increased level of glutamate in the retina will cause neurotoxic effects and the activation of ionotropic glutamate receptors in excess, mainly the N-methyl-D-aspartate receptor (NMDAR), which results in cell death by mechanisms such as uncontrolled intracellular calcium responses [73,74,75] (Figure 4).

In detail, the accumulation of glutamate in retinal cells contributes to cell death in many types of cells via various mechanisms as Figure 4 shows. The retina is a simple layered evagination of the brain, located at the back of the eye. It can not only detect light but also encode the light signals into electrical ones and transmit the message to the brain to “see” objects. In addition to photoreceptors, the main retinal cells are horizontal, bipolar, amacrine, Müller, and ganglion cells.

In the retinal ganglion cells, the excessive extracellular concentration of glutamate results in an uncontrolled continuous depolarization of neurons, as a toxic process called excitotoxicity. In excitotoxicity, glutamate triggers the increase of intracellular Ca^2+^ levels, and this process is predominantly mediated by NMDA-type glutamate receptors (NMDARs) in RGCs isolated from neonatal or adult rats. AMPA/kainate-Rs (AMPAR/KAR) also contribute a smaller portion of the Ca^2+^ response at saturating concentrations of glutamate [75,76,77]. After the increase of intracellular Ca^2+^, there follows an upregulation of nNOS (neuronal nitric oxide synthase), dysfunction of mitochondria, reactive oxygen species (ROS) production, ER stress, and release of lysosomal enzymes [78,79,80], all of which lead to excitotoxic, oxidative, and apoptotic stress. Excessive Ca^2+^ concentration is the key mediator of glutamate toxicity through the over-activation of ionotropic and metabotropic receptors [78]. In addition, after the activation of nuclear factor kB (NF-kB) in Müller cells, the production of endogenous glia-derived TNF-α increases the RGC surface levels of Ca^2+^-permeable AMPA receptors (AMPAR) and triggers cellular death, which is mediated by the insertion of Ca^2+^-permeable AMPAR into RGC membrane [81,82]. Glutamate accumulation can also inhibit the uptake of cystine (CySS) by reversing the action of the CySS/glutamate antiporter. Reversal of the antiporter action reinforces the aforementioned events by depletion of cysteine and eventually glutathione’s reducing potential [78]. Glutamate has been shown to increase the generation of nitric oxide (NO) [83], whose interactions with oxygen radicals are reported to mediate a glutamate-induced delayed death of retinal neurons in retinal ischemia [84]. All these can accelerate the process of RGC death.

Studies have been conducted in other types of cells, as well. For instance, the incubation of bovine retinal endothelial cells with glutamate increases both NO and oxidative stress [85]. In addition, in normal Müller cells, the glutamate transporter (GLAST) mediates the transport of glutamate into cells, after which glutamate becomes glutamine by the effect of glutamine synthetase and moves out of Müller cells mediated by Na^+^-coupled neutral amino acid transporters (SNATs) in the form of glutamine to presynaptic cells. However, if GLAST function impairs, perhaps resulting from oxidative stress caused by other processes, extracellular glutamate will increase, and NMDARs will be activated, following the overproduction of NO and increase of oxidative stress [85,86,87]. Finally, cellular functions are impaired as a consequence.

In addition, inflammation is recognized as a critical driver of the retinopathy process. Glutamate and homocysteine are both differentially expressed metabolites in the studies of metabolomics, and they can both induce inflammatory responses in the retina.

Fan et al. [88] found that glutamate activates distinct NF-κB proteins in the retina, and the activation of a member in the NF-κB family, P65, may be especially important with regard to retinal ganglion cell (RGC) responses to glutamate because its activity is induced by conditions that are known to result in the death of these cells. The NMDA receptor-Ca^2+^-CaMKII signaling pathway is involved in this glutamate-induced NF-κB activation. Elsherbiny et al. [89] indicated that hyperhomocysteinemia induces inflammatory responses in the mouse retina. In detail, mice with hyperhomocysteinemia due to a lack of the enzyme cystathionine-β-synthase (CBS) and wild-type mice were evaluated for microglia activation and inflammatory markers. In addition, human retinal endothelial cells and retinal pigment epithelial (RPE) cells treated with/without homocysteine were evaluated for inflammatory cytokines and NF-κB activation using the multiplex assay, Western blot analysis, and immuno-fluorescence. NF-κB was activated and cytokine array analysis showed a marked increase in pro-inflammatory cytokines and downregulation of anti-inflammatory cytokines. Singh and Tyagi [90] used gene microarray analyses on RPE cells treated with homocysteine. Alterations in the expressions of several inflammatory gene transcripts are revealed. The transcripts for CCL5, CEBPB, IL13RA2, IL15RA, IL6, IL8, and CXCL3 were up-regulated while the transcripts for C3, CCL2, IL11RA, and IL18 genes exhibited down-regulation. The IL6 and CEBPB expressions were subsequently validated at the protein levels. Glutamate and homocysteine, differentially expressed in the metabolomics of DR, AMD, and ROP, induce inflammatory responses in the retina.

## 3. Conclusions

To gain insight of vision impairment and blindness caused by retinopathy, we summarize the metabolism of amino acids in retinopathy with the analysis of metabolomics and conclude the differential amino acids and related metabolic pathways for biomarker and therapy of DR, AMD, and ROP.

In terms of the potential as biomarkers, different amino acids have different characteristics, leading to various clinical applications. Glutamine and arginine tend to increase in peripheral blood samples from DR, AMD, and ROP patients according to the analysis of metabolomics [17,18,19,20,21,23,25,26,27,28,29,43,45,52,55,56,58], which makes it possible and reasonable for glutamine and arginine to be the biomarkers used in early diagnosis of retinopathy. In addition, the level of alanine rises in DR patients in intraocular fluids, blood samples, and even in CSF and urine while not showing clear tendency in AMD and ROP [19,22,27,31,32,33]. This distinction between diseases leads to the possibility of alanine being the specific biomarker for DR. Meanwhile, there are also therapeutic benefits. Methionine decreases in blood samples of DR patients and histidine decreases in the plasma of AMD patients, but intraocular changes are not discovered in both disorders, indicating that the supplement of certain amino acids may become a therapeutic measure and be of benefit to patients. What is more, besides the aforementioned potential as biomarkers, glutamine may act as a target for DR treatment because it increases in almost all types of samples of DR patients including plasma, serum, vitreous humor, aqueous humor, and urine. By decreasing the glutamine levels in DR patients, there may be an unexpected improvement. These results may allow the development of metabolic biomarkers for early diagnosis and novel therapeutic strategies to prevent or delay the development of DR, AMD, and ROP.

In conclusion, this review delineates the significance of amino acid metabolism in main retinopathy and provides possible direction to discover therapeutic targets for retinopathy, which is of great importance clinically.

## Figures and Tables

**Table 1:** Summary of amino acids in diabetic retinopathy based on metabolomics studies.

Species	Samples	Subjects	Platforms	Criteria	Differential Metabolites	Study
Human	Plasma	42 DR/ 32 NDR	UPLC-MS	Fold change (FC) > 1.2 and *p* < 0.05	N-Fructosyl isoleucine	Sun et al. (2021) [17]
					N-acetyltryptophan	
					Leucylleucine	
					Kynurenic acid	
					3-Methylhistidine	
					Phenylacetylglutamine	
		21 PDR/ 21 NPDR			Glutamine	
		52 PDR/ 72 NPDR/ 59 NDR	UPLC-MS, GC-MS	Variable important in the projection (VIP) > 0.7	asparagine, aspartic acid, glutamic acid, glutamine, glycine, methionine, pyroglutamic acid	Rhee et al. (2018) [18]
		21 PDR/ 21 NDR	UPLC-MS	*p* < 10 × 10^−5^, Area under the curve (AUC) ≥ 0.95, VIP > 1	L-serine, β-alanine, L-Proline, L-Homoserine,4-Hydroxyproline, Ornithine, L-Aspartic acid, L-Glutamine, L-Glutamic acid, L-Methionine, N-Acetylornithine, L-Arginine, N-Acetyl-L-aspartic acid, Citrulline, Phosphoserine	Zhu et al. (2019) [19]
		83 DR/ 90 NDR	LC-MS	VIP ≥ 2.0	arginine, citrulline	Sumarriva et al. (2019) [20]
		64 PDR/ 92 NPDR/ 159 NDR	LC-MS	FDR (false-discovery rate) < 0.05	↑: arginine, citrulline (DR/ NDR)	Peters et al. (2021) [21]
		19 DR/ 14 controls	NMR	*p* < 0.05	↑: leucine, isoleucine, tyrosine, and valine; ↓: histidine, Alanine	Lin et al. (2019) [22]
		88 PDR/ 51 controls	UPLC-MS	VIP > 1, FC > 1.2 and < 0.83, FDR = 0.05	↑: Pyroglutamic acid, Alpha-N-phenylacetyl-L glutamine	Wang et al. (2022) [23]
	Serum	25 NDR/ 39 DR/ 25 PDR	GC-MS	largest VIP	L-aspartic acid	Li et al. (2011) [24]
		176 DR/ 329 NDR	LC-MS	*p* < 0.05	↑: asymmetric dimethylarginine (ADMA), L-arginine, symmetric dimethylarginine (SDMA)	Abhary et al. (2009) [25]
		689 DR/ 216 control	GC-MS, LC-MS	*p* < 0.05, FDR < 0.05	↑: serine, glycine, arginine, ornithine, citrulline, proline, leucine, isoleucine, and valine	Xuan et al. (2020) [26]
		51 PDR/ 123 NPDR/ 143 NDR	LC–MS	*p* < 0.05	DR/ NDR: proline, NPDR than NDR: alanine, aspartic acid, and glutamine, arginine, histidine, lysine, methionine, threonine, tryptophan, and tyrosine, PDR than NDR: lysine, methionine, serine, tryptophan, and tyrosine; PDR than NPDR: total dimethylarginine	Yun et al. (2020) [27]
		69 DR/ 69 NDR	UPLC-MS	VIP > 1, FC< 0.8 or > 1.2 and FDR < 0.05	ornithine, phenylacetylglutamine	Zuo et al. (2021) [28]
		123 DR/ 116 NDR	Metabolon DiscoveryHD4	*p* < 0.05	cysteine-glutathione disulfide, phenylacetylglutamine, cys-gly (oxidized), N-acetylmethionine glycylvaline, phenylalanine, aspartate, tryptophan, glutamate	Yousri et al. (2022) [29]
	Plasma and serum	228 PDR/ 276 NPDR/ 141 NDR	GC-MS, UHPLC-MS	(Benjamini–Hochberg) BH- adjusted *p* < 0.05	alanine and serine	Curovic et al. (2020) [30]
		666 DR/ 2211 NDR	NMR	*p* < 0.05	tyrosine	Quek et al. (2021) [31]
	Vitreous humor	28 PDR/ 22 NDM	GC-MS	VIP > 1 *p* < 0.05	alanine, alloisoleucine, creatinine, glutamine, leucine, lysine, ornithine, pyroglutamic acid, phenylalanine, threonine, valine	Wang et al. (2020) [32]
		51 PDR/ 23 controls	UPLC-MS	VIP > 1 FC >1.2 and < 0.83 FDR = 0.05	alpha-N-phenylacetyl-L glutamine, pyroglutamic acid	Wang et al. (2022) [23]
		22 PDR/ 22 NDM	NMR	*p* ≤ 0.05	alanine, valine, glutamine, leucine, isoleucine	Barba et al. (2010) [33]
		35 PDR/ 19 NDM	UHPLC-MS	*p* < 0.05	citrulline, dimethylglycine, glycine, ornithine, proline, creatine	Tomita et al. (2021) [34]
		20 PDR/ 31 NDM	HPLC-MS	*p* < 0.05	Methionine, arginine, proline, citrulline, ornithine	Paris et al. (2016) [35]
		9 PDR/ 8 controls	UHPLC-MS	*p* < 0.05	citrulline, glutamine, N-amidino-L-aspartate, proline, 5-oxoproline	Haines et al. (2018) [36]
	Aqueous and humor	13 DR/ 14 NDR	NMR	VIP > 1.0 *p* < 0.05	asparagine, DMA, glutamine, histidine, threonine	Jin et al. (2019) [37]
		23 PDR/ 25 NDM	GC-MS	VIP > 1.0 *p* < 0.05	citrulline	Wang et al. (2020) [32]
	Aqueous and vitreous humor	18 PDR/ 22 controls	LC-MS	*p* < 0.05	Cysteine persulfides (CysSSH), cystine, oxidized glutathione trisulfide (GSSSG)	Kunikata et al. (2017) [38]
	CSF	19 DR/ 14 controls	NMR	FC > 1.2 or < 0.8, FDR < 0.05	alanine, leucine, isoleucine, tyrosine, and valine, histidine,	Lin et al. (2019) [22]
	Urine	666 DR/ 2211 NDR	NMR	*p* < 0.05	Alanine, glutamine	Quek et al. (2021) [31]
	Fecal samples	21 PDR/ 14 NDR	UPLC-MS	VIP > 1, *p* < 0.05	pyro-L-glutaminyl-L-glutamine, D-proline, N-gamma-L-glutamyl-D-alanine, N-acetyl-L-methionine, L-threo-3-phenylserine,	Zhou et al. (2021) [39]
		45 PDR/ 90 NDR	UPLC-MS	*p* < 0.05, VIP> 1, and log2 FC > 1	tyrosine	Ye et al. (2021) [40]
Rats	Urine	6 DR rats/6 controls	UPLC-MS	VIP > 1, *p* < 0.05	phenylacetylglycine, 5-l-glutamyl-taurine	Wang et al. (2020) [41]
Zebrafish	Whole body	50 pdx1−/− zebrafish	UHPLC–MS	*p* < 0.05	glutamate, proline, ornithine, tyrosine	Wiggenhauser et al. (2020) [42]

DR, diabetic retinopathy; NDR, no diabetic retinopathy (with diabetes without diabetic retinopathy); NDM, no diabetes; PDR, proliferative diabetic retinopathy; NPDR, non-proliferative diabetic retinopathy; GC-MS, gas chromatography mass spectrometry; LC-MS, liquid chromatography–mass spectrometry; HPLC-MS, high-performance liquid chromatography–mass spectrometry; UPLC-MS, ultra-performance liquid chromatography–mass spectrometry; UHPLC-MS, ultra-high-performance liquid chromatography–mass spectrometry; NMR, nuclear magnetic resonance; Metabolon, DiscoveryHD4, DiscoveryHD4™, Metabolon, UK; CSF, cerebrospinal fluid.

**Table 2:** Summary of amino acids in age-related macular degeneration based on metabolomics studies.

Species	Samples	Subjects	Platforms	Criteria	Differential Metabolites	Study
Human	Plasma	Coimbra: 201 AMD /42 controls	NMR	*p* < 0.05	Early AMD vs. Controls: creatine; Late vs. Intermediate AMD: histidine	Laíns et al. (2017) [43]
		Boston: 113 AMD /40 controls		*p* < 0.05	Early AMD vs. Controls: glutamine; Intermediate vs. Early AMD: glutamine, histidine; Late vs. Intermediate AMD: histidine, alanine	
		91 IAMD /100 NVAMD /195 controls	LC-MS	VIP ≥ 2.0 and an *p* < 0.05	AMD vs. controls: pyroglutamic acid; NVAMD vs. IAMD: kynurenine	Mitchell et al. (2021) [44]
		20 wetAMD /20 controls	UHPLC-MS, QTOF-MS	VIP > 1 and *p* < 0.05 or 0.05 < *p* < 0.1	N-Acetyl-L-alanine, L-Tyrosine, L-Phenylalanine, L-Methionine, L-Arginine,	Luo et al. (2017) [45]
		Boston: 149 AMD /47 controls, Coimbra: 242 AMD /53 controls	UHPLC-MS	FDR < 0.05	Beta-citrylglutamate, N-acetylmethionine, aspartate, N-acetylasparagine, S-adenosylhomocysteine (SAH), isoleucylglycine N-acetylasparagine, beta-citrylglutamate, N-acetylleucine	Laíns et al. (2019) [46]
		26 NVAMD /19 controls	LC-FTMS	FDR< 0.05	Acetylphenylalanine	Osborn et al. (2013) [47]
		32 AMD /32 controls	HPLC-MS	*p* < 0.05	Homocysteine	Ghosh et al. (2013) [48]
		40 AMD /40 controls	LC-MS	0.6 < FC < 1.4 *p* < 0.1	Valine, lysine, proline	Chao et al. (2020) [49]
		127 wAMD /50 controls	UHPLC-MS	FC ≥ 2 and FC ≤ 0.5, *p* < 0.05, VIP ≥ 1	L-Tryptophan; L-Alanyl-L-Lysine	Deng et al. (2021) [50]
	Plasma	53 AMD /18 controls	UPLC-MS	*p* < 0.01	N-acetylglutamine, N-acetylleucine	Mendez et al. (2021) [51]
	Serum	72 AMD /72 controls	microLC-MS	sPLSda (to select the most predictive variables from dataset)	(Predictors for non-advanced AMD) glutamine, glutamate:glutamine ratio, glutaminolysis	Kersten et al. (2019) [52]
	Aqueous humor	26 wetAMD /20 controls	UHPLC-MS	VIP > 1.0 and *p* < 0.05	N6, N6, N6-trimethyl-L-lysine, norleucine, L-phenylalanine, γ-glutamylglutamine, N-acetylhistidine, creatine, N-fructosyl isoleucine, L-proline	Han et al. (2020) [53]
	Urine	Coimbra cohort: 252 AMD /53 controls	NMR	*p* < 0.05	Late AMD vs. Intermediate AMD: valine	Laíns et al. (2019) [54]
		Boston cohort: 147 AMD /47 controls		*p* < 0.05	Intermediate AMD vs. Early AMD: glycine, lysine, tyrosine	

Int., intermediate AMD; LC-MS, liquid chromatography–mass spectrometry; HPLC-MS, high-performance liquid chromatography–mass spectrometry; UPLC-MS, ultra-performance liquid chromatography–mass spectrometry; UHPLC-MS, ultra-high-performance liquid chromatography–mass spectrometry; NMR, nuclear magnetic resonance; micro LC-MS, microflow liquid chromatography–tandem mass spectrometry; Q-TOF, Quadrupole-Time of Flight; LC-FTMS, liquid chromatography with Fourier-transform mass spectrometry; RIT, rod-intercept time (under dark adaption); AUDAC, area under the dark adaption curve (under dark adaption).

**Table 3:** Summary of amino acids in retinopathy of prematurity based on metabolomics studies.

Species	Samples	Subjects	Platforms	Criteria	Differential Metabolites	Study
Human	Plasma	58 ROP /25 controls	HPLC-MS/MS	*p* < 0.05	Creatinine, citrulline, arginine, and aminoadipic acid, creatinine, and aminoadipic acid	Zhou et al. (2021) [55]
		38 treatment requiring-ROP /23 ROP	UHPLC-MS	FC > 1.5, *p* < 0.05	L-Lysine, L-Citrulline, Pro-Thr, L-Glutamine, L-Pyroglutamic acid, L-Tryptophan, Cysteine-S-sulfate,	Zhou et al. (2020) [56]
		57 ROP /57 controls	UPLC-MS/MS	VIP > 0.5	Glutamic acid gamma-methyl ester, Picolinoylglycine, Creatinine, Ornithine, 1-Carboxyethylisoleucine	Yang et al. (2022) [57]
		26 ROP /29 controls	LC-MS/MS	*p* < 0.05	Arginine, Lysine, aspartic acid, glutamine	Ozcan et al. (2020) [58]
	Serum	87 ROP	NMR	*p* < 0.05	Phenylalanine, Lysine	Nilsson et al. (2022) [59]
	Heel blood	40 ROP /41 controls	UPLC-MS	VIP > 1	Glycine, glutamate, leucine, serine, valine, tryptophan, citrulline and homocysteine	Yang et al. (2020) [60]
OIR mice	Retina	10 OIR /10 controls	HPLC-MS/MS	FC > 1.5, *p* < 0.05.	Dimethylglycine, L-Citrulline, N-Acetyl-L-aspartic acid, L-Alanine, Gly-Glu, D-Proline, Gamma-L-Glutamyl-L-glutamic acid, Creatine, L-Pyroglutamic acid, L-Leucine, L-Aspartate, L-Glutamate, Glycine, L-Citrulline, D-Proline, L-Valine, N-Acetyl-L-aspartic acid ↓, Gamma-L-Glutamyl-L-glutamic acid, Creatine, L-Alanine, L-Aspartate	Zhou et al. (2021) [61]
	Whole eyes	3 P12 mice /3 P14 mice /4 P17 mice /5 controls	HPLC-MS	*p* < 0.05	Arginine, Proline, Citrulline, Ornithine, Lysine	Paris et al. (2016) [35]
Rats	Plasma	Four groups, each with 12 individuals: (i) CT1; (ii) HO1; (iii) CT2; (iv) HO2	GC-MS	*p* < 0.05, VIP ≥ 1	HO1/CT1: L-Tyrosine, L-Proline, Ornithine, L-Tryptophan, L-Glutamine, L-Threonine, 4-Hydroxy-L-proline, Glycine, HO2/HO1: L-Proline, L-Alanine, L-Glutamine, L-Tyrosine, Ornithine, L-Threonine, L-Valine, L-Alloisoleucine, L-Lysine, L-Serine, L-Histidine, L-Arginine	Lu et al. (2020) [62]

GC-MS, gas chromatography mass spectrometry; LC-MS, liquid chromatography–mass spectrometry; HPLC-MS, high-performance liquid chromatography–mass spectrometry; UPLC-MS, ultra-performance liquid chromatography–mass spectrometry; UHPLC-MS, ultra-high-performance liquid chromatography–mass spectrometry; NMR, nuclear magnetic resonance; P12 mice, mice harvested at twelve days after birth; CT1, reared in room air and sampled at P12; HO1 (simulating the vaso-obliteration process (phase I)), exposed to high oxygen for 5 days and sampled at P12; CT2, reared in room air and sampled at P17; HO2 (simulating the neovasculization one (phase II)), exposed to high oxygen for 5 days then followed by room air for 5 days and sampled at P17.
